# A Dark Incubation Period Is Important for *Agrobacterium*-Mediated Transformation of Mature Internode Explants of Sweet Orange, Grapefruit, Citron, and a Citrange Rootstock

**DOI:** 10.1371/journal.pone.0047426

**Published:** 2012-10-17

**Authors:** Mizuri Marutani-Hert, Kim D. Bowman, Greg T. McCollum, T. Erik Mirkov, Terence J. Evens, Randall P. Niedz

**Affiliations:** 1 U.S. Horticultural Research Laboratory, Agricultural Research Service, U.S. Department of Agriculture, Ft. Pierce, Florida, United States of America; 2 Department of Plant Pathology and Microbiology, Texas A&M University, Texas AgriLIFE Research, Weslaco, Texas, United States of America; Kansas State University, United States of America

## Abstract

**Background:**

Citrus has an extended juvenile phase and trees can take 2–20 years to transition to the adult reproductive phase and produce fruit. For citrus variety development this substantially prolongs the time before adult traits, such as fruit yield and quality, can be evaluated. Methods to transform tissue from mature citrus trees would shorten the evaluation period via the direct production of adult phase transgenic citrus trees.

**Methodology/Principal Findings:**

Factors important for promoting shoot regeneration from internode explants from adult phase citrus trees were identified and included a dark incubation period and the use of the cytokinin zeatin riboside. Transgenic trees were produced from four citrus types including sweet orange, citron, grapefruit, and a trifoliate hybrid using the identified factors and factor settings.

**Significance:**

The critical importance of a dark incubation period for shoot regeneration was established. These results confirm previous reports on the feasibility of transforming mature tissue from sweet orange and are the first to document the transformation of mature tissue from grapefruit, citron, and a trifoliate hybrid.

## Introduction

Citrus is grown worldwide, is consumed both fresh and processed, and is one of the most economically important fruit crops. Broadly viewed, new citrus rootstock and scion varieties are developed for three general reasons: 1) improve resistance to pest, disease, and environmental problems to which current commercial rootstock and scion cultivars are susceptible; 2) increase fruit/juice yield to improve profitability to growers; and 3) improve fruit/juice quality to remain competitive in the marketplace. The development of new citrus rootstock and scion varieties takes several decades and can span the careers of multiple researchers. One of the reasons for the long development time is an extended juvenile phase in citrus that typically requires at least 5–10 years before flowering. Another reason is that a new selection is tested at multiple field locations, with multiple rootstocks or scions, and over at least four years of fruit production to provide an estimate of productivity.

Plant breeders develop new varieties by introducing genetic variability, generally through controlled hybridizations, into a crop and selecting individuals with useful characteristics. However, there are some serious barriers in citrus biology that must be overcome before progress can be made, and include: the difficulties and expense of working with a tree crop, a long juvenile phase, and many citrus exhibit apomixis and inbreeding depression. These barriers, taken together, make citrus one of the most difficult crops to breed. Genetic engineering is one method to introduce genetic variability into citrus that can potentially address those problems in citrus that have no known viable long-term solution. For example, Huanglongbing (HLB) or citrus greening disease is considered the most serious citrus disease in the world [1]. However, because there is little natural resistance to HLB, it will be difficult to develop resistant varieties through conventional plant breeding methods. Recombinant DNA approaches provide a potential solution for developing citrus trees resistant to HLB and, more generally, many of the serious problems that either threaten or seriously limit citrus production and that have no management or conventional breeding solutions.

**Figure 1:**
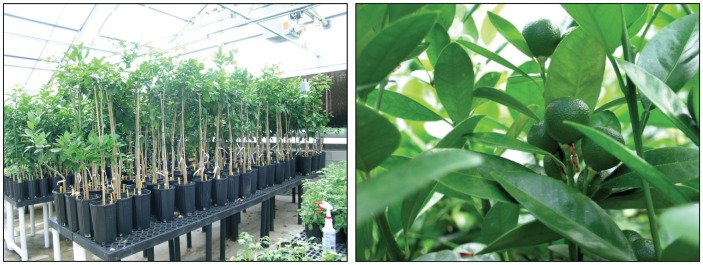
Four populations of greenhouse-grown adult phase citrus trees were used: Valencia sweet orange, Ruby Red grapefruit, US942 citrange rootstock, and Etrog citron. Ruby Red grapefruit trees with fruit are shown.

**Figure 2:**
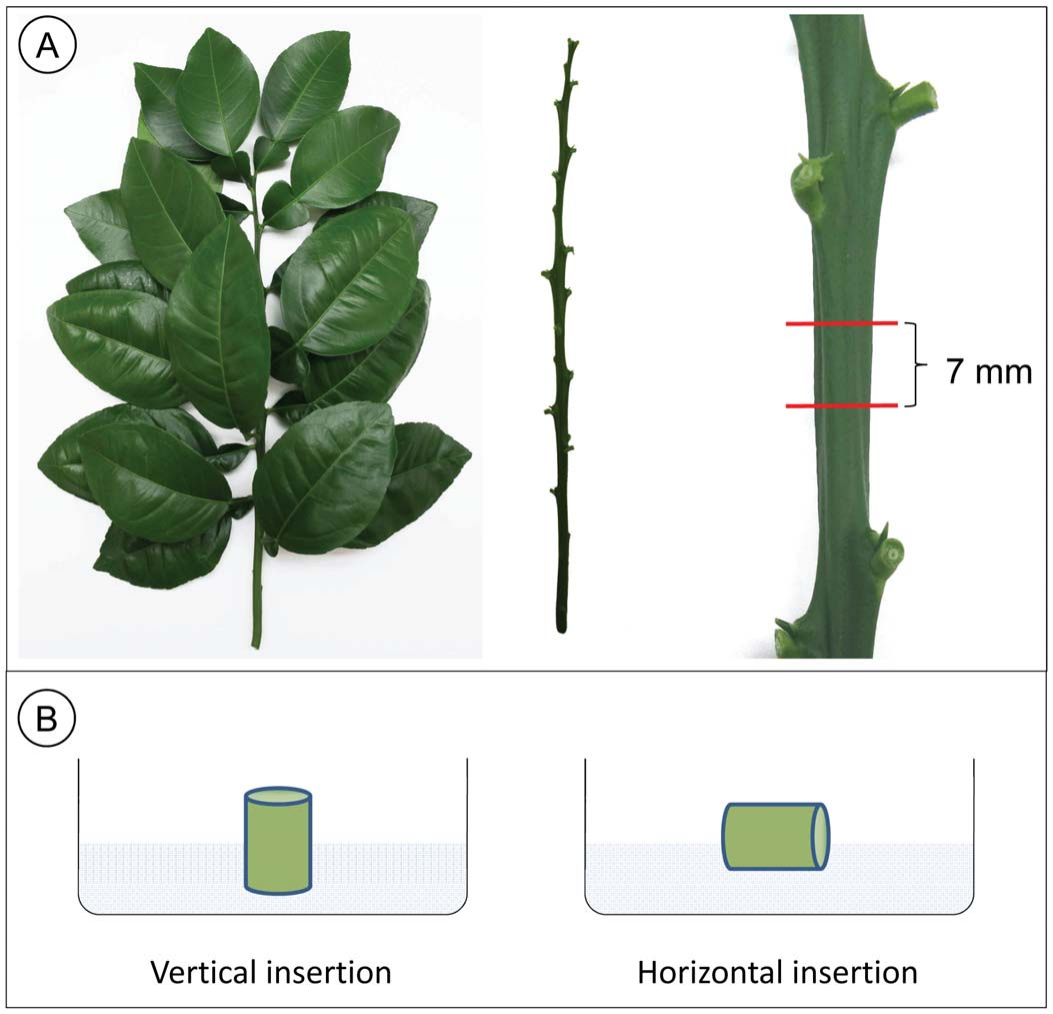
Internode explants. A) The source of internode explants were new fully expanded shoots approximately 30 cm in length. Leaves were removed prior to disinfestation. Internode explants were excised as 7 mm segments. B) Internode explants were inserted into the culture media vertically for shoot regeneration experiments and horizontally for transformation experiments.

Genetic transformation of citrus rootstock and scion varieties is an active area of research and methods to transform citrus have been reported for many of the major citrus types, including sweet orange [2]–[7], mandarin [8], [9], grapefruit [10]–[13], lemon [14], [15], lime [16], [17], and important rootstock types [18]–[20]. However, because the primary methods used to transform citrus use juvenile tissue, seedlings or embryogenic cell lines, transgenic citrus trees have the same long juvenile phase as trees grown from seed. To circumvent the juvenile phase requires the transformation of tissue from mature trees.

Mature tissue transformation in citrus has been reported for sweet orange [4], [21]–[23] and mandarin [9]. In these studies, factors examined for their effect on mature tissue transformation included flush, *Agrobacterium* strain, and feeder plates [21]; BAP singly or in combination with NAA [9], [22], [23], variety of sweet orange [22], the use of a helper plasmid containing additional copies of virG, virE1, and virE2 genes [9], the concentration of 2,4-D in the co-cultivation medium and the length of co-cultivation [9].

**Table 1:** Design points for the AgNO_3_-GA-PGR-Basal media experiment.

Design Points	Factors				Response
	AgNO_3_ mg/L	GA µM	PGR	Basal media	Shoot number
1	2.5	5	10 µM ZR	WPM	1.625
2	2.5	1.25	15 µM BA +10 µM NAA	MS	0
3	5	5	10 µM ZR	MS	7.5
4	0	5	15 µM BA +10 µM NAA	WPM	0
5	0	2.5	15 µM BA +10 µM NAA	MS	0
6	5	0	15 µM BA +10 µM NAA	MS	0.143
7	2.5	0	10 µM ZR	MS	4.5
8	0	5	10 µM ZR	WPM	0.625
9	0	5	10 µM ZR	MS	9.667
10	1.25	2.5	15 µM BA +10 µM NAA	WPM	0
11	5	0	10 µM ZR	WPM	1
12	2.5	1.25	10 µM ZR	WPM	1
13	5	0	15 µM BA +10 µM NAA	MS	0
14	0	0	15 µM BA +10 µM NAA	MS	0.125
15	0	0	10 µM ZR	WPM	1.125
16	5	5	10 µM ZR	MS	6.25
17	5	5	15 µM BA +10 µM NAA	WPM	0
18	2.5	0	15 µM BA +10 µM NAA	WPM	0.125
19	2.5	5	15 µM BA +10 µM NAA	MS	0.25
20	5	5	15 µM BA +10 µM NAA	WPM	0
21	1.25	2.5	10 µM ZR	MS	4.5
22	5	0	10 µM ZR	WPM	1.75
23	0	5	10 µM ZR	MS	8.5

The experiment is a 4-factor response surface design with sufficient points for a quadratic model. Shoot number is the mean response from eight culture dishes, each containing a single internode explant. Replicated points included #11/#22, #17/#20, #9/#23, #3/#16, and #6/#13. ANOVA presented in Table 3.

For plant breeding applications, genetic transformation systems should be 1) sufficiently efficient to produce the required numbers of independent transgenics per transgene to alter the phenotype of the targeted trait(s) while retaining the integrity of the variety and, 2) applicable to a broad range of types, commercial and breeding, within the crop. Our objectives were twofold – first, to determine the effects of factors potentially important (dark incubation, AgNO_3_, GA, basal medium, and growth regulators) for shoot regeneration from mature tissue, a necessary prerequisite for improving the efficiency of mature tissue transformation and, second, to broaden the range of citrus types transformable from mature tissue by including three citrus types not previously transformed including grapefruit, citron, a trifoliate hybrid rootstock, and a high quality and important sweet orange variety (Valencia) not previously reported.

**Table 2:** Effect of dark incubation on the number of shoots regenerated from sweet orange, grapefruit, and US-942 internode explants.

Source	Sweet orange	Grapefruit	US-942
	Prob > F	Prob > F	Prob > F
*Weeks in the dark*	<0.0001	<0.0001	0.0007
One Sample T-Test			
*0 Weeks = 0*	–	–	–
*1 Weeks = 0*	0.0610	–	–
	(0.47±0.51)		
*2 Weeks = 0*	<0.0001	0.0003	0.0100
	(9.04±5.48)	(5.29±3.12)	(1.99±0.10)
*3 Weeks = 0*	<0.0001	<0.0001	n/a^a^
	(2.85±0.44)	(4.18±1.16)	
*4 Weeks = 0*	0.0085	0.0010	0.0013
	(0.44±0.15)	(2.29±1.71)	(1.22±0.87)
*6 Weeks = 0*	0.1834	0.0003	0.0023
	(0.27±0.38)	(2.57±1.24)	(1.05±0.43)
*8 Weeks = 0*	–	0.0034	0.0012
		(0.75±0.35)	(1.58±1.43)

ANOVA p-values (Prob. > F) for the effect of dark incubation on shoot regeneration in sweet orange, grapefruit, and US-942. One sample t-tests p-values that compared each dark incubation period mean for each citrus type was compared to zero. The number of shoots is presented as the mean ± standard deviation from two experiments that included 9−17 internode explants. Cells with no p-value were all zero (i.e., zero variance) and could not be tested.

a US-942 did not include a three week test.

**Figure 3:**
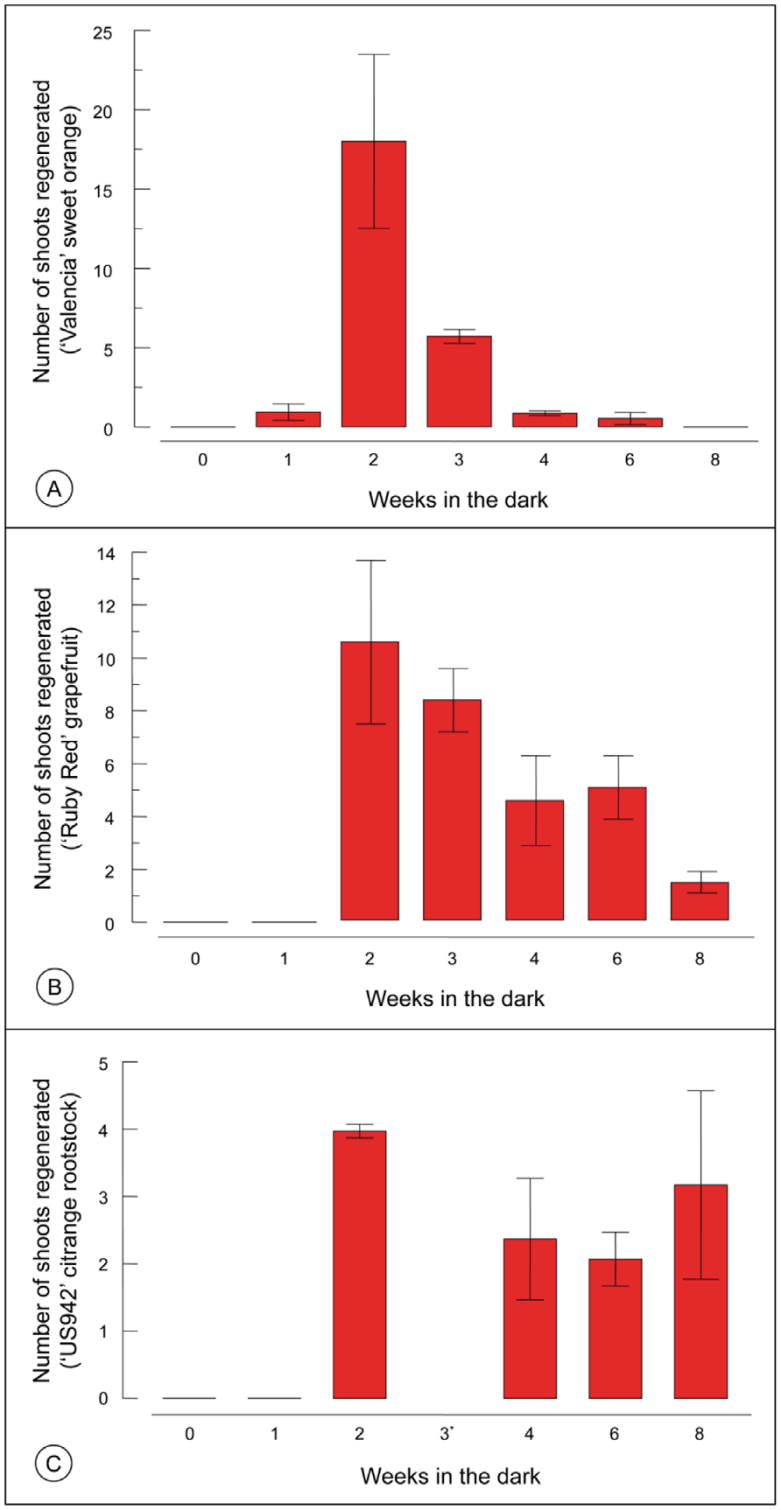
Bar graphs of the effect of the dark incubation period on the number of shoots regenerated from sweet orange, grapefruit, and US942 internode explants. Explants were incubated in the dark for 0 to 8 weeks prior to culture in a 16/8 photoperiod for three weeks. Data are presented as mean ± standard deviation. US942 did not include a three week dark treatment.

## Materials and Methods

### Source Plants and Explant Preparation

*Citrus paradisi* Macf. Ruby Red (grapefruit), *C. sinensis* (L.) Osbeck Valencia (sweet orange), *C. medica* (L.) Etrog (citron), and US-942 [24] rootstock (*C. reticulata* Sunki×*Poncirus trifoliata* Flying Dragon) adult phase trees (i.e., had produced flowers) maintained under greenhouse conditions were used as the source of internode explants (Figure 1). New fully expanded shoots (30 cm long) were used as the source of internodes. After removing the leaves, the shoots were surface sterilized by the following sequence – washed in running water for 5 mins, immersed for 15 min in 5 g/L Alconox® (Alconox, Inc. White Plains, NY) soapy water, 30 sec in 70% ethanol, 30 min in 1% sodium hypochlorite solution and Tween 20 (3 drops/L), and then three rinses in sterile distilled water [25]. Explants approximately 7 mm in length were prepared from the internodes (Figure 2A) using sterile garden scissors. Internode explants prepared in this manner were used in the dark incubation, shoot regeneration, and *Agrobacterium*-mediated transformation experiments.

**Table 3:** Effect of AgNO_3_, GA, PGR, and basal medium on numbers of shoots regenerated from mature intermodal explants of US-942.

Effects	df	F Value	p-values
Model	8	88.95	<0.0001
*Main effects*			
AgNO_3_	1	0.35	0.564
GA	1	0.14	0.7173
PGR	1	382.17	<0.0001
Media	1	66.9	<0.0001
*2-way interaction effects*			
GA×Media	1	10.78	0.0054
PGR×Media	1	18.63	0.0007
*Curvature effects*			
AgNO_3_^2^	1	3.83	0.0705
GA^2^	1	12.39	0.0034
Lack of Fit	p = 0.7198		
R^2^	0.98		
R^2^ adjusted	0.97		
R^2^ predicted	0.95		
Model type^a^	reduced quadratic		
Transformation^b^	Log_10_		

ANOVA p-values (Prob. > F) and summary statistics for the experimental design and data from Table 1.

aModel reduction by backward elimination.

b Data log10 transformed per Box Cox analysis. Log_10_(Shoot # +0.097).

**Figure 4:**
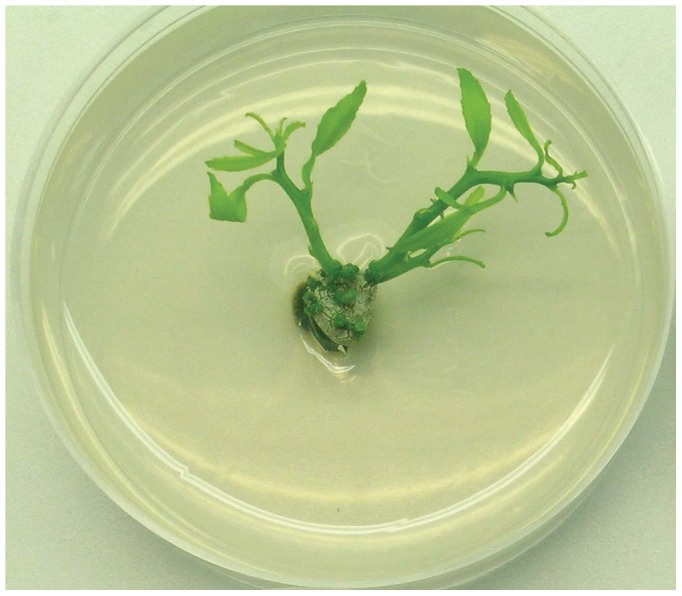
Shoot regeneration from US942 internode explant–design point #3/#16 (MS +10 µM ZR +5 mg/L AgNO3+5 µM GA).

### Dark Incubation Experiment

The effect of a dark incubation period on shoot regeneration from internodes of sweet orange, grapefruit, and US-942 was determined. The experiment was designed as a single factor design. Each internode explant was inserted vertically (Figure 2B) into a Falcon #351007 60×15 mm polystyrene dish (Becton Dickinson Labware, NJ, USA) containing 10 mL shoot induction medium (SIM) composed of MS salts [26] (PhytoTechnology Laboratories®, Shawnee Mission, KS, USA), glycine (1 mg/L), thiamine-HCl (1 mg/L), pyridoxine-HCl (1 mg/L), nicotinic acid (1 mg/L), zeatin riboside (10 µM) (Gold Biotechnology, St. Louis, MO, USA), 0.8% (w/v) agar (USB Corporation, OH), and the pH reading adjusted to 5.7 with NaOH. Internode explant cultures from sweet orange, grapefruit, and US-942 were incubated in a dark chamber (27°C) for 0, 1, 2, 3, 4, 6, or 8 weeks, then transferred to 16/8 h photoperiod, 35 µmol m^−2^ s^−1^ at 27°C for 21 days, and then data were collected (buds/shoots counted). The experiment was repeated and the response at each treatment point was estimated from 9–17 internode explants per replicate. The number of buds/shoots produced was counted and the data analyzed by one-way ANOVA for each citrus type followed by a one-sample t-test that compared the mean of each treatment to 0 (i.e., no buds/shoots produced).

**Table 4:** Individual experiment and summary statistics for US-942 regeneration and transformation results.

Citrus type	Number of internodes cultured	Number of shoots regenerated	Number of GUS^+^ shoots	Regeneration (%)	Transformation efficiency (%)^a^
US-942	20	16	1	80.00	5.00
US-942	62	5	2	8.06	3.23
US-942	65	5	2	7.69	3.08
US-942	202	31	3	15.35	1.49
US-942	100	36	7	36.00	7.00
Summary^b^	449	93	15	29.42±30.53	3.96±2.11

a Transformation efficiency was calculated as the number of GUS positive plants/number of internode explants.

b Presented as totals or mean ± standard deviation.

**Figure 5:**
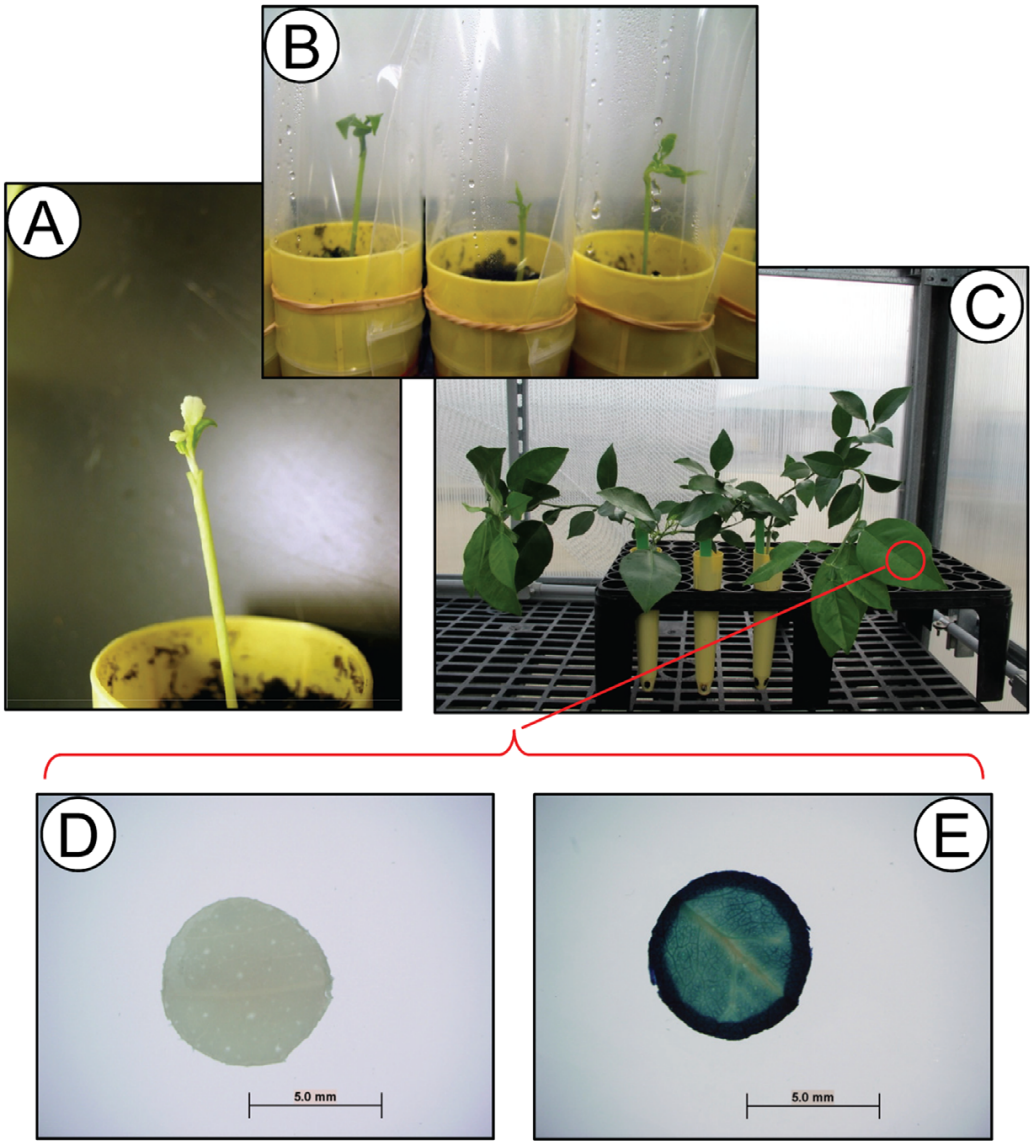
Transition from *in vitro* to *ex vitro* growth. A) *In vitro* shoots from *Agrobacterium*-treated internode explants were micrografted on to *ex vitro* grown seedling rootstock, B) micrografted shoots were covered with a plastic bag and grown in a growth chamber (27°C, 55 µmol m-2 s-1, 16-h photoperiod) for two weeks, and then C) moved to the greenhouse. Vigorously growing plants were tested for GUS activity by X-Gluc histochemical staining circular explants punched out from the midrib of a leaf – D) GUS negative, E) GUS positive.

### AgNO_3_-GA-PGR-Basal Media Experiment

Four factors considered important or potentially important in shoot regeneration were examined using US-942 and included AgNO_3_ (0 to 5 mg/L), GA (0 to 5 µM), PGR (10 µM ZR or 15 µM BA and 10 µM NAA), and basal medium (MS or WPM). A four-factor response surface design was constructed that included two numeric factors, AgNO_3_ and GA, and two categorical factors, PGR and basal medium. Design points were selected using D-optimal criterion, modified to include lack-of-fit points, sufficient to estimate a quadratic polynomial. The experimental design included 13 model points, 5 lack-of-fit points, and 5 points to estimate pure error, i.e. 23 design points. The design points are listed in Table 1. The number of shoots regenerated at each design point was estimated from eight 60×15 mm culture dishes (184 total), each containing a single internode explant inserted vertically (Figure 2B) into SIM modified per the specified treatment. Internode explant cultures were incubated in a dark chamber (27°C) for 2 weeks, then transferred to 16/8 h photoperiod, 35 µmol m^−2^ s^−1^ at 27°C for 21 days, and then data were collected (buds/shoots counted). Data were analyzed by analysis of variance (ANOVA).

**Table 5:** Individual experiment and summary statistics for Valencia sweet orange regeneration and transformation results.

Citrus type	Number of internodes cultured	Number of shoots regenerated	Number of GUS^+^ shoots	Regeneration (%)	Transformation efficiency (%)^a^
sweet orange	22	0	0	0	0
sweet orange	118	19	0	16.10	0
sweet orange	240	18	1	7.50	0.42
sweet orange	100	9	2	9.00	2.00
sweet orange	100	13	2	13.00	2.00
Summary^b^	580	59	5	9.12±6.12	0.88±1.03

a Transformation efficiency was calculated as the number of GUS positive plants/number of internode explants.

b Presented as totals or mean ± standard deviation.

**Figure 6:**
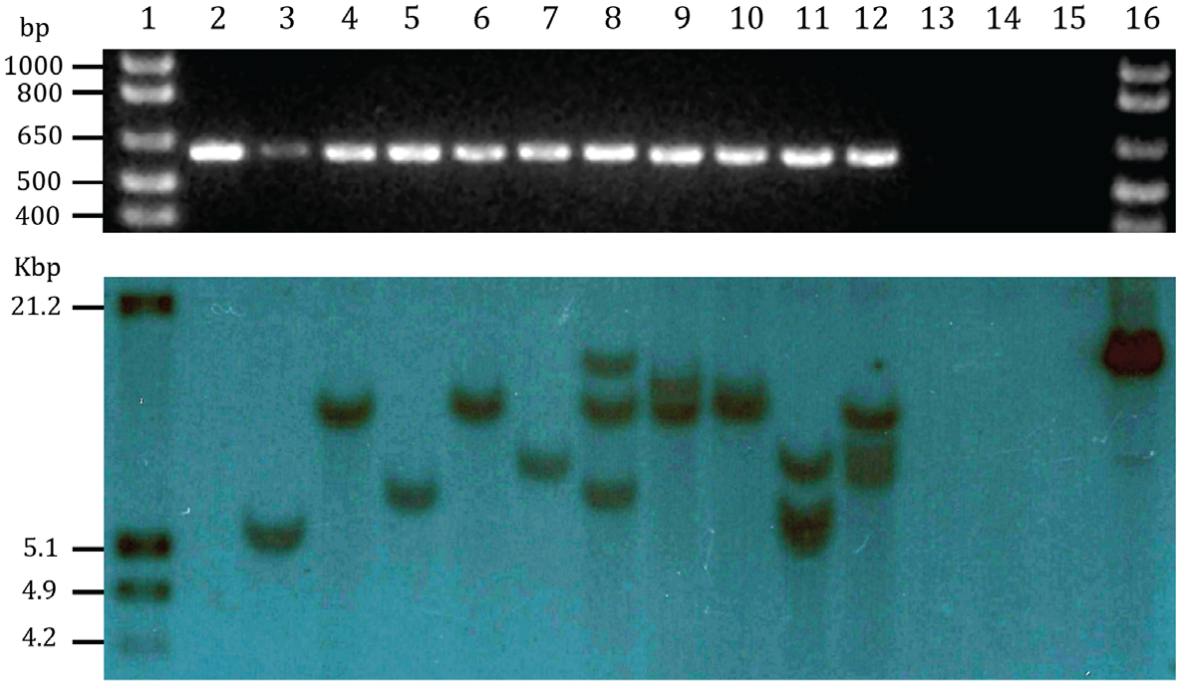
PCR gel (top image) and Southern blot (bottom image) analyses of 10 independent GUS-positive citrus transformants. Lane 1– molecular weight markers; Lane 2– Binary plasmid p35S GUS INT [28] used in transformation experiments; Lanes 3 and 4– transgenic ‘Valencia’ sweet orange; Lanes 5–8– transgenic ‘Etrog’ citron; Lanes 9–12– transgenic US-942 rootstock (*C. reticulata* ’Sunki’ ×*Poncirus trifoliata* ‘Flying Dragon’); Lane 13– Untransformed ‘Etrog’ citron; Lane 14– Untransformed ‘Valencia’ sweet orange; Lane 15– Untransformed US-942. Lane 16– molecular weight markers.

### Agrobacterium-mediated Transformation

*Agrobacterium tumefaciens* strain EHA-105 strain [27] carrying the p35S GUS INT binary plasmid [28] carrying the marker gene β-glucuronidase (GUS) was used in all experiments. Bacterial cultures were made by selecting a single colony from a streaked YEP plate (10 g/L peptone, 5 g/L NaCl, 10 g/L yeast extract, 10 g/L agar, and pH 7), and inoculating a 125 ml flask containing 50 mL of liquid YEP + kanamycin (1 mg/ml) + acetosyringone (40 µM), and culturing on a rotary shaker (225 rpm) at 27°C until the culture achieved an OD_600_ of 1.3 to 1.5. The *Agrobacterium* inoculation solution was prepared by diluting the *Agrobacterium* culture with SIM to a final OD_600_ of 0.5, and then adding acetosyringone to 40 µM.

**Table 6:** Individual experiment and summary statistics for Etrog citron regeneration and transformation results.

Citrus type	Number of internodes cultured	Number of shoots regenerated	Number of GUS^+^ shoots	Regeneration (%)	Transformation efficiency (%)^a^
citron	25	4	0	16.00	0.00
citron	44	4	0	9.09	0.00
citron	121	18	7	14.88	5.79
citron	121	6	2	4.96	1.65
citron	120	3	0	2.50	0
Summary	431	35	9	9.49±5.94	1.49±2.51

a Transformation efficiency was calculated as the number of GUS positive plants/number of internode explants.

b Presented as totals or mean ± standard deviation.

**Table 7:** Individual experiment and summary statistics for Ruby Red grapefruit regeneration and transformation results.

Citrus type	Number of internodes cultured	Number of shoots regenerated	Number of GUS^+^ shoots	Regeneration (%)	Transformation efficiency (%)^a^
grapefruit	19	6	1	31.58	5.26
grapefruit	50	1	0	2.00	0
grapefruit	104	6	0	5.77	0
grapefruit	87	6	0	6.90	0
grapefruit	124	9	0	7.26	0
grapefruit	100	7	0	7.00	0
Summary^b^	484	35	1	10.70±11.86	1.05±2.35

a Transformation efficiency was calculated as the number of GUS positive plants/number of internode explants.

b Presented as totals or mean ± standard deviation.

Approximately one hundred internode explants were prepared at a time. As internode explants were excised they were placed into SIM liquid medium. Once the explants were prepared the SIM medium was discarded and replaced with *Agrobacterium* inoculation solution for 15 minutes, after which the explants were blotted dry with paper towels. Internode explants were inserted horizontally (Figure 2B) into 100 x 20 mm culture dishes, 20 explants per dish, containing co-culture medium composed of SIM medium with 40 µM acetosyringone, and incubated at 24°C in the dark for three days.

Following co-culture, explants were removed and triple rinsed with water in a 500 mL glass bottle and then blotted dry with paper towels. Explants were then placed, one explant per dish, into 60 ×15 mm culture dishes containing SIM +5 µM GA + antibiotics (100 mg/L kanamycin, 250 mg/L vancomycin, and 250 mg/L cefotaxime), incubated in the dark at 27°C for two weeks, transferred to 16/8 h photoperiod, 35 µmol m^−2^ s^−1^ at 27°C for 30 days for 3–4 weeks, and subcultured monthly thereafter.

### Micro-grafting of GUS (β-glururonidase) Positive Shoots

Regenerated shoots >2 mm were grafted onto rootstock seedlings. Valencia, Ruby Red, and Etrog were grafted onto US-812 [29] citrus rootstock (*C. reticulata* ’Sunki’ ×*Poncirus trifoliata* Benecke) and regenerated shoots of US-942 were grafted onto *C. volkameriana* Ten. & Pasq. (Volkamer).

#### Rootstock preparation

A rootstock seed was planted in PRO MIX BX soil (Premier tech Horticulture, Quakertown, PA) in a 164 ml Ray Leach Cone-tainer cell SC10 (Stuewe & Sons, Inc, Tangent, OR). PRO MIX BX soil was steamed at 71°C for 2 h 15 min before use. Rootstocks were grown etiolated at 27°C in the greenhouse for a month. When the rootstock seedlings were approximately 10 cm tall, they were used for micro-grafting with transformed shoots. Before grafting, the seedlings were fed with ¼ Basal Salt Mixture (Murashige & Skoog, PhytoTechnology Laboratories, Shawnee Mission, KS) and also given two eye droplets to each pot of a solution of Sequestrene 138, Iron Chelate Mix, 1.2 g/L (Becker Underwood, Inc., Ames, IA.).

**GUS assay.** Regenerated shoots >2 mm were excised from internode explants and the bottom 1–2 mm of the base of the shoot was excised for assay for GUS activity. The basal sample was placed into a 1.5 microcentrifuge tube containing 200 µL GUS assay buffer [30] and incubated overnight at 37°C. The shoot was inserted into a 100×20 mm culture dish containing SIM. Shoots where the basal sample turned blue via the X-gluc histochemical assay were then micrografted.

#### Micrografting procedure

Regenerated shoots >2 mm were shaped to look like the alphabet “V” at the base of the shoot. Rootstocks were cut under the cotyledon and sliced vertically in the middle of the stem with a Double Edge Carbon Steel Breakable Razor Blade (Electron Microscopy Sciences, Hatfield, PA). Trimmed shoots were inserted into the vertical incision. Immediately after grafting, the Cone-tainer pot was covered with a plastic bag and sealed with a rubber band to retain moisture. Grafted plants were incubated for one week in a growth chamber programmed to a 16/8 photoperiod and 27°C and then transferred to the greenhouse.

### PCR Analysis and Southern Hybridizations

#### PCR analysis

DNA was extracted from transformed GUS^+^ trees and GUS^−^ nontransformed trees using DNase Plant Mini Kits (Qiagen, Valencia, CA, USA) per the manufacturer’s directions. The *uidA* gene was amplified with 5′GAATGGTGATTACCGACGAAA3′ and 5′CCAGTCGAGCATCTCTTCAGC3′ designed to amplify a 574 bp fragment [31]. PCR was performed in a MJ Research thermocycler and included 34 cycles of denaturation at 94°C for 1 min, annealing at 65°C for 1 min, and extension at 72°C for 2 min. Each sample included 60 ng of DNA and was separated by 100 volt electrophoresis in 1.0% agarose gels, and visualized by ethidium bromide staining.

#### Southern hybridizations

Total DNA was extracted from leaf tissue following the method of Dellaporta [32]. DNA extracts were treated with RNase, phenol-chloroform extracted, and ethanol precipitated; pellets were dissolved in Tris-EDTA and DNA was quantified using a nanodrop spectrophotometer (Thermo Scientific, Wilmington, DE). Each DNA extract was digested with EcoRI; 5 µg of each EcoRI-digested DNA extract was separated by electrophoresis in 0.8% agarose and subsequently transferred to a nylon membrane (Roche Applied Science, Indianapolis, IN) following the manufacturer’s instructions. A PCR product was generated using the transformation vector (p35S GUS INT) as template along with the same primers used for confirmation of *uidA* transgene insertion. The GUS PCR product served as template for the preparation of a DIG-labeled hybridization probe using the DIG DNA Labeling Kit (Roche Applied Science) following the manufacturer’s instructions. All subsequent hybridization, stringency washes, and detection steps were carried out using the DIG detection kit (Roche Applied Science) following the manufacturer’s instructions.

## Results

### Dark Incubation Experiment

The effect of a dark incubation period on the number of shoots regenerated was determined for sweet orange, grapefruit, and US-942; citron was not tested. For all three citrus types shoots were only observed with a dark incubation period; no shoots were observed for the 0 week dark treatment. The SIM medium used was based on results from prior experimentation that determined the effects of various cytokinins and auxins on shoot regeneration from juvenile epicotyl explants of sweet orange and grapefruit [33]. Shoots were counted three weeks after the explants were moved from dark to light. An ANOVA was conducted for each citrus type and showed that the effect of a dark incubation period on the number of shoots regenerated was significant (Table 2); the means ± S.D. are shown in Table 2 and Figure 3. A one sample t-test was used to determine which dark incubation treatments for each citrus type had shoot numbers greater than 0. The one sample t-test and 0 values were used because no shoots were regenerated in the 0 week dark treatment. For sweet orange, 2–4 weeks in the dark produced a significantly greater number of shoots than 0; for grapefruit and US-942 it was 2–8 weeks. The three week treatment was not run with US-942.

### AgNO_3_-GA-PGR-Basal Medium Experiment

The number of shoots was counted three weeks after the explants were moved to light. Shoot number for US-942 ranged from 0 to 9.6 (Table 1) and indicated that AgNO_3_, GA, PGR, and basal medium affected shoot number (Figure 4). A summary of the ANOVA, lack-of-fit test and three R^2^ statistics for quality are presented in Table 3. A reduced quadratic polynomial model was selected (p<0.0001). Data was transformed with a log10 function as the Box Cox plot identified a violation of the normality assumption. Residual and model diagnostics were within acceptable limits. The lack-of-fit test was not significant (p = 0.7198) and indicated that additional variation in the residuals could not be removed with a better model. R^2^, R^2^_adj_ and R^2^_pred_ statistics ranged from 0.98–0.95. Main effects were the primary affecters of shoot regeneration. PGR had the single largest effect on the number of shoots regenerated (F Value = 382; p<0.0001) and was due to the large positive effect of zeatin riboside. The type of basal medium was also highly significant (F Value = 67; p<0.0001), though much less so than PGR, with more shoots regenerated on MS medium than WPM. The two-way interaction effects of GA and PGR with basal medium were significant. Quadratic effects (i.e., curvature of the response) were significant for GA. Based on these results the treatment selected for use in the transformation experiment was MS +5 µM GA +10 µM ZR (replicated design point #9/#23).

### Transformation Experiments

Twenty-one transformation experiments were conducted with the four citrus types (Tables 4–7) and resulted in a total of 30 transgenic shoots identified by GUS staining, including 15 US-942, 5 Valencia sweet orange, 9 Etrog citron, and 1 Ruby Red grapefruit. Transformed trees were obtained by micrografting (Figure 5) from sweet orange, citron and US-942– the grapefruit graft was lost. Transgenic plants obtained from micrografting were reconfirmed by the expression of GUS in leaf discs (Figure 5), PCR amplification of the *uidA* gene fragment and Southern blot analysis using a *uidA* gene fragment probe (Figure 6).

## Discussion

Because shoot regeneration is an essential prerequisite for *Agrobacterium*-mediated transformation experiments, the basic approach was to first identify factors important for promoting shoot regeneration from internode explants from adult phase citrus trees, and then to produce transgenic trees using the identified factors and factor levels for inducing shoots. To a large extent, the citrus types and numbers of explants used in the various experiments was subject to the type and quantity of plant material available at the time. Internode explants were inserted vertically for the shoot regeneration experiments for convenience; because shoots only developed on the upper surface, their formation and development were more easily monitored than explants placed horizontally. Horizontal placement was required for transformation experiments to expose tissue to the selective antibiotic kanamycin.

Extensive preliminary experiments on the effects of various growth regulators on shoot regeneration resulted in a small number of internode explants developing small shoot primordia that never developed into shoots (unpublished data). Because these experiments utilized only a 16/8 photoperiod (i.e., no dark incubation), the effect of a dark incubation period was considered. For juvenile citrus tissue, a dark incubation period enhanced shoot regeneration in sweet orange [34] and lime [16], but was inhibitory in citrange [35]. For mature citrus tissue, the effect of a dark incubation period has not been determined. Of the five studies that report transformation of mature citrus tissue, four included a dark incubation period [9], [21]–[23] as part of the protocol. The single study that did not include a dark incubation period [4] utilized adventitious buds that formed directly from the wounded regions on seedlings where the growing tip and axillary buds were removed. Of the four studies that report shoot regeneration from mature citrus tissue, three include dark incubation [36]–[38] and one did not [39]. Though a dark incubation period is often included in transformation and shoot regeneration protocols, the periods are variable and range from 15 [21] to 50 days [38]. These results suggested genotype or condition-specific effects and, therefore the importance of testing a dark incubation period under local conditions when initiating a citrus tissue culture project. We observed that a dark incubation period was essential for shoot regeneration from sweet orange, grapefruit, and US-942; citron was not tested. When internode explants were incubated in the dark, they developed numerous buds and shoots; no shoots were regenerated from any of the citrus types without a dark incubation period. We also observed that shoot regeneration generally declined after a 2 week dark period, but this may not be true for transformation. Cervera et al. [9] extended the dark incubation period from 2–4 weeks to 5–6 weeks to promote more callus formation, with the intent of improving transformation efficiency; however, the specific effect of this change on transformation was not determined. The cytokinin zeatin riboside was used in these dark experiments as it was the most effective cytokinin for shoot regeneration from juvenile epicotyl explants of ‘Valencia’ sweet orange and worked well for ‘Duncan’ grapefruit [33]; it was selected on the assumption that it might also work well with mature tissue. To the best of our knowledge, all citrus mature tissue shoot regeneration and transformation experiments utilize BAP alone or in combination with NAA to induce shoots.

To further improve shoot regeneration, the individual and combined effects of four additional factors identified as potentially important were determined. The protocol for this experiment included a two week dark incubation period per the results from the dark incubation study. AgNO_3_, an ethylene inhibitor, was selected because it can sometimes enhance shoot regeneration [40]–[41]. Because its effect on shoot regeneration was weak, AgNO_3_ was not included in the shoot regeneration medium used for transformation. GA was selected as a potential enhancer of shoot regeneration and growth [36], [43]–[45] and, because its effect was small but positive on shoot regeneration, GA was included in the shoot regeneration medium used for transformation. BA + NAA was compared to ZR as BA + NAA are two growth regulators commonly used together to regenerate shoots from mature citrus explants [9], [22], [23], [46]. MS and WPM were selected as they have both been used in citrus tissue culture [9], [36], [46]. The use of ZR was the most important factor that affected shoot regeneration and indicates the importance of using the appropriate cytokinin. The level of ZR used in this experiment was 10 µM, a level found to be useful for juvenile explants [33] and therefore potentially not optimal for mature internode shoot regeneration. In addition, because the growth regulator treatments were fixed concentrations, their effects on mature tissue shoot regeneration over a range of proportions and concentrations could not be determined. To fully explore these effects would require a 3-component (ZR-BA-NAA) mixture-amount experimental design. Given the large effect of ZR, additional research to optimize growth regulator types, proportions, and concentration for mature tissue may provide further improvements. This argument also applies to the comparison of MS and WPM where little is learned, apart from which formulation works better, about the proportions and concentrations of specific mineral nutrients and their effects on shoot regeneration.

Transgenic plants were obtained by *Agrobacterium*-mediated transformation of internode explants from mature greenhouse grown trees of sweet orange, grapefruit, citron, and a trifoliate hybrid rootstock. The results confirm previous reports on the feasibility of transforming mature tissue from sweet orange and are the first to document the transformation of mature tissue from grapefruit, citron, and a trifoliate hybrid. Variability was high and was presumably due to the various relatively uncontrolled environmental and physiological effects. For example, the transformation experiments were conducted over an eight-month period where plant material was harvested from greenhouse-grown trees exposed to light levels and seasonal temperatures that varied widely. Also, the type and condition of the shoot flush can affect regeneration frequencies [21]; internode explants were taken from stems harvested from trees from which shoot flushes were previously harvested. These factors and their interaction on transformation efficiency are essentially unknown, not accounted for, and therefore part of the observed variability.

Transformation efficiency was greatest for US-942. This may be because trifoliate hybrids are some of the most transformable citrus types. However, the AgNO_3_-GA-PGR-Basal medium experiment was conducted only on US-942 and the identified conditions for good shoot regeneration were then applied to all the citrus types. To what extent this effect was due to the inherent responsiveness of trifoliate hybrids vs. using conditions specifically optimized for US-942 is unknown.

In this study we have quantified the effects of a dark incubation period and the medium components AgNO_3_, GA, type of PGR, and basal medium on shoot regeneration from internode explants from adult phase greenhouse-grown citrus trees. Under our conditions, a dark incubation period was essential and GA, zeatin riboside, and MS basal medium enhanced shoot regeneration. The assumption that treatments that enhanced shoot regeneration would also enhance transformation was made and four diverse citrus were transformed using a single protocol. Because transgenic plants were obtained from these four citrus types, with little control of the initial physiological status of the trees, suggests 1) the potential importance of optimizing protocols to local conditions and, 2) that further improvements in transformation efficiencies across a broad range of citrus types are likely.
